# Bone Densities Assessed by Hounsfield Units at L5 in Computed Tomography Image Independently Predict Hepatocellular Carcinoma Development in Cirrhotic Patients

**DOI:** 10.3390/jcm11195562

**Published:** 2022-09-22

**Authors:** Christopher Yeh, Ming-Wei Lai, Chau-Ting Yeh, Yang-Hsiang Lin, Jeng-Hwei Tseng

**Affiliations:** 1 School of Medicine, California University of Science and Medicine, Colton, CA 92324, USA; 2 Liver Research Center, Chang Gung Memorial Hospital, Linkou, Taoyuan 33305, Taiwan; 3 Department of Radiology, Chang Gung Medical Center, Taoyuan 33305, Taiwan

**Keywords:** hepatocellular carcinoma, bone density, computed tomography, prognosis

## Abstract

A previous study identified that bone density (BD) assessed by Hounsfield unit (HU) at T12 in computed tomography (CT) image was a predictor for hepatocellular carcinoma (HCC) development in cirrhotic patients. Here, we conducted a verification study, where clinical variables together with BDs (assessed from three different bone areas: T12, L5, and femur trochanter) were assessed for their predictive values for time-to-HCC development in cirrhotic patients. Univariate Cox proportional hazard analysis showed that age (*p* = 0.017), T12 BD (*p* = 0.013) and L5 BD (*p* = 0.005), but not femur BD, were significant predictors. Multivariate analysis revealed that L5 BD was the only independent factor associated with time-to-HCC development (adjusted *p* = 0.007). Kaplan-Meier analysis confirmed that BD which was lower than median HU was associated with a shorter time-to-HCC development for both T12 BD and L5 BD (*p* = 0.001 each). Longitudinal follow-ups for BDs in HCC patients having received serial CT imaging studies unveiled a significantly rapid reduction in BD, right before HCC was diagnosed (*p* = 0.025 when compared with the average BD reduction rate). In conclusion, BD assessed by HU at L5 was an independent predictor for HCC development in cirrhotic patients. Rapid BD reduction during CT scan follow-ups could serve as a warning sign for HCC development.

## 1. Introduction

Hepatocellular carcinoma (HCC) is an aggressive cancer with a mean survival time varying from 6 to 20 months if untreated [1]. A great proportion of patients with HCC are in far advanced stages, carrying portal vein occlusion/invasion or distant metastasis, and have an even shorter survival time. Early detection and diagnosis are the key steps toward improving patients’ survival, since only early-stage HCC can be completely resected or ablated by surgical or non-surgical measurements [2]. The major etiological factors of HCC are chronic hepatitis B/C and alcoholism, as well as other causes of chronic hepatitis, which could ultimately lead to liver cirrhosis, before development of malignancy [1,3]. The existing standard methods for HCC detection include alpha fetal protein (AFP) monitoring and liver ultrasonography [4]. During the period of HCC surveillance, the patients have often received many imaging studies for cross confirmation, allowing researchers to develop novel HCC risk predictors.

One of the leading projects assimilating digital algorithms into the clinical world is analytic morphomics [5]. This method aims to extract multiple digital features from standard computed tomography (CT) scans and provides statistics that accurately describe and predict patients’ conditions and outcomes. The features that can be incorporated into a predictive algorithm include measurements describing organ conditions, muscle qualities, visceral and subcutaneous fat qualities, cortical and trabecular bone densities, and vascular characteristics.

Previous studies have utilized analytic morphomics, attempting to stratify patients’ risk of HCC based on the predictive models being built [5]. In particular, trabecular bone density (BD) as well as other body compositions, has been found to be a predictor for the development of HCC in cirrhotic patients as well as for posttransplant survival in HCC patients [6,7,8,9,10]. In the cirrhotic study, trabecular BD was a predictor for HCC development in 126 Taiwanese patients (*p* = 0.044) and in 274 American patients (*p* = 0.030) [7]. In the posttransplant study, bone mineral density was an independent predictor (*p* = 0.030) for posttransplant survival in 118 patients receiving liver transplantation [10]. Many theories can be proposed for this association, such as vitamin D deficiency, bridging between osteoporosis to increased risk of HCC. However, the value of predicting algorithms lies in its intrinsic trait of not requiring a thorough mechanistic explanation before valuable clinical application can be implemented.

Despite these intriguing findings, the *p* value for the cirrhotic study was marginal in the Taiwanese population, possibly owing to a smaller sample size [7]. In this study, we aimed to validate the result of this study with a larger independent cohort of samples and to assess the trabecular BD measurements obtained from three different bone areas. Furthermore, we examined whether there was a progressive change in BD during the longitudinal course toward HCC.

## 2. Materials and Methods

### 2.1. Patients

Under the approval of institutional review board, Chang Gung Memorial Hospital, Taoyuan, Taiwan, clinical data and CT images of 347 patients diagnosed as liver cirrhosis (from 2005 to 2014) were retrospectively retrieved form our electronic chart record. These CT scans were originally taken for evaluation of cirrhotic nodules or surveys for other diseases. Of the 347 cirrhotic patients included, 311 belonged to this group. For the remaining 36 patients, CT scan was performed for other non-liver diseases. HCC was not found in the CT images included in this study. The CT images which had been used in the previous risk score finding study [7] were excluded. Clinical data including age, sex, Child-Pugh classification, hepatitis B virus (HBV) surface antigen (HBsAg), antibody against hepatitis C virus (anti-HCV), alcoholism, aspartate transaminases (AST), alanine transaminase (ALT), alpha-fetoprotein (AFP), white blood cell (WBC) count, hemoglobin, platelet count, and trabecular bone densities assessed by CT scan were recorded. The time when the CT scan was taken was recorded; the time when HCC was diagnosed or the time of last follow-up was also recorded. Diagnosis of HCC was made by either (1) aspiration cytology (2) histology by biopsy, or (3) dynamic CT or MRI imaging study with typical HCC characteristics, if tumor > 1 cm was found in cirrhotic patients.

### 2.2. Evaluation of Trabecular BDs

Trabecular BDs were evaluated by use of CT derived Hounsfield Units (HUs) as previously described [7]. However, HUs were evaluated for three areas in the present study: T12, L5 and femur trochanter areas.

### 2.3. Statistical Methods

Parametric parameters with normal distribution were presented as mean ± standard deviation (SD). Otherwise, they were presented as median (range). Dichotomized parameters were presented as number (%). Student’s *t* test was used to compare parametric parameters with normal distribution. Otherwise, the Mann-Whitney test was applied. A Chi-Square test with yates’ correction or Fisher’s exact test was used to compare dichotomized variables where appropriate. Univariate and multivariate Cox proportional hazard model was used to evaluate factors associated with time-to-HCC development. The Kaplan-Meier analysis was used to confirm the results. *p* < 0.05 was consider statistically significant.

## 3. Results

### 3.1. Baseline Characteristics of Patients

A cohort of 347 cirrhotic patients, who had at least one CT scan image taken before HCC was diagnosed, were enrolled. Since this was an independent verification study, CT scans that had been used in the previous risk score finding study were excluded. The baseline characteristics of patients were listed in Table 1. The majority of patients were male (67.4%), aged 58.9 ± 11.3 years. Of them, 87.9% had Child-Pugh classification A. A great proportion (64.0%) was HBV related. Since the baseline data were obtained when HCC had not yet been detected, the AFP levels were low (median: 3.3 ng/mL).

Of all of the patients included, 25 patients had baseline AFP > 20 ng/mL, with 4 of them > 100 ng/mL. Of these 25 patients, 11 had developed HCC in the later follow-ups, but all occurred > 3 months (6.5 months to 152.0 months) after the baseline CT imaging was performed. Of these 11 patients, 9 had documented hepatitis exacerbations when the baseline AFP was assayed. Therefore, the elevated AFP was likely due to hepatitis flares but not HCC. For the remaining 2 patients, the AFP levels were 50.0 ng/mL and 93.5 ng/mL, respectively, and HCC was diagnosed 22.4 months and 25.4 months, respectively, after the baseline CT images were taken. The cause of high AFP in these two patients were not clear. In this study, all HCCs occurred at least 3 months after the baseline CT scan was performed. 

This was a retrospective study, the hemogram data were not routinely checked at the time of CT imaging study. Missing data were noted for WBC, hemoglobin, and platelet (see Table 1 footnote). During the median follow-up period of 52.3 months (range, 3 to 223.4 months), 93 (26.8%) patients developed HCC. The BDs were assessed from three areas of the CT images: T12, L5, and femur trochanter. The HU values from T12 and L5 were similar (means, 175.3 and 172.8, respectively), whereas the value was lower (mean, 91.1) for data from femur trochanter. Notably, 4 CT images did not have femur BD data, because the femur bone was not the focus of their diseases under study.

HBsAg, hepatitis B virus surface antigen; anti-HCV, antibody against hepatitis C virus; AST, aspartate transaminase; ALT, alanine transaminase; AFP, alpha-fetoprotein; WBC, white blood cell; BD, bone density; HU, Hounsfield unit; HCC, hepatocellular carcinoma.

### 3.2. Associations between the Three BDs Measured

To understand the association between the three BD assessments, linear regression analysis and Pearson’s correlation coefficient were performed (Table 2 and Table 3). It was found the BD assessed from T12 was highly associated with that from L5 and femur (*p* < 0.001 each). Additionally, it was tightly associated with age (*p* < 0.001). 

### 3.3. Clinical Factors and Bone Densities in Relation to Time-to-HCC Development

Subsequently, we examined the association between the clinical variables and time-to-HCC development (Table 4). Univariate Cox proportional hazard analysis was performed. It was found that among all clinical factors collected, only age (*p* = 0.017), T12 BD (*p* = 0.013), and L5 BD (*p* = 0.005) were associated with time-to-HCC development. Notably, femur BD was not significantly associated with time-to-HCC development. To understand whether BD was a risk factor independent of age, we performed a multivariate analysis. Under the stepwise mode, it was found that only L5 BD was remained in the multivariate model (adjusted *p* = 0.007). When L5 BD and age were included for multivariate analysis using enter mode, it was found that only L5 BD was only borderline associated with time-to-HCC development (adjusted *p* = 0.077). However, if L5 BD HU less than 161 was used as a predictor for HCC (see the later section for optimal cutoff), and this predictor and age were included for multivariate analysis, it was found that L5 BD HU less than 161 was a predictor, independent to age (adjusted *p* = 0.041). These data suggested that L5 BD was an independent predictor for HCC development.

**Figure 1:**
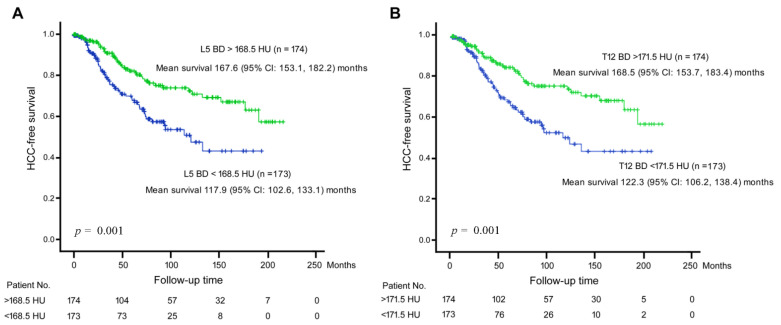
Kaplan-Meier analysis for BD in association with time-to-HCC development. (**A**) BD assessed by HU at L5 area. The median HU value (168.5 HU) was used as a cutoff. Green line, L5 BD > 168.5 HU; Blue line, L5 BD < 168.5 HU. (**B**) BD assessed by HU at T12 area. The median HU value (171.5 HU) was used as a cutoff. Green line, T12 BD > 171.5 HU; Blue line, L5 BD < 171.5 HU. Vertical axis, HCC-free survival rate; Horizontal axis, follow-up time period.

To further verify our finding, a Kaplan-Meier analysis was performed for L5 BD and T12 BD in association with time-to-HCC development (Figure 1). By using the median HU values as cutoffs, it was found that L5 BD and T12 BD were both significantly associated with HCC development.

### 3.4. Sequential Changes in Bone Densities before HCC Development

**Figure 2:**
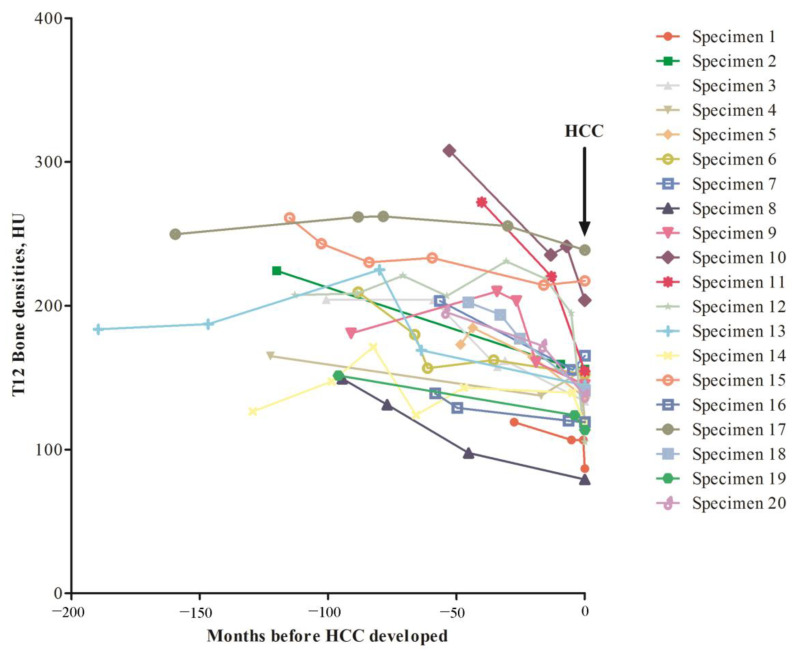
Retrospective examination of BD changes in serial CT images of 20 patients diagnosed as HCC. Arrow, time point when HCC was diagnosed. Vertical axis, T12 BD in HU unit; Horizontal axis, time period before HCC was diagnosed.

Subsequently, we examined serial BD changes before and upon HCC development. In our patients, some of them had serial CT scan images taken. Here, we randomly included 20 patients who had at least 3 CT scan images taken before and upon HCC had been diagnosed. The BD assessed from T12 was performed for all images (Figure 2). It was discovered that the BD fluctuated before development of HCC. The BD reduction rate of these 20 patients was 0.79 ± 0.68 HU/month when all the serial BD HU values were included for calculation. However, if the BD reduction rate was calculated using the last two BDs (right before and upon HCC diagnosed), then the reduction rate was 4.7 ± 8.6 HU/month. A comparison between these two rates showed that *p* = 0.025 (Paired Wilcoxon Signed Ranks test). In fact, only two patients (patient No. 3922571 and 3747891) had mild elevation of BD before and upon HCC was diagnosed. Taken together, a significantly increased of BD reduction rate was found before HCC was diagnosed.

### 3.5. Positive and Negative Predictive Values for L5 BD HU < 161 to Predict HCC Occurrence

Finally, we evaluated the predictive values of L5 BD to predict HCC occurrence within definite periods of time. Of all patients, 9, 22, and 44 patients developed HCC within 1, 2, and 3 years of follow-ups. The L5 BD values were (HCC versus non-HCC) 166.7 ± 35.9 versus 173.0 ± 49.1 (*p* = 0.621; HCC within 1 year), 154.3 ± 30.2 versus 174.1 ± 49.6 (*p* = 0.008; HCC within 2 years), and 159.3 ± 37.1 versus 174.8 ± 50.0 (*p* = 0.016; HCC within 3 years), respectively. By use of Receiver operating characteristic curve method, the optimal cutoff of L5 BD was 161.0. The positive/negative predictive values were 3.2%/97.9%, 9.0%/95.8% and 16.7%/90.6%, respectively, for HCC occurring within 1, 2, and 3 years. Using L5 BD HU < 161 as a predictor for HCC, multivariate analysis including this predictor and age showed that it was an independent predictor (Table 4). 

## 4. Discussion

One of the most challenging aspects of “standardizing” care is the interference from each clinician’s initial subjective evaluation. Even with vigorous research attempting to establish standard guidelines, many subjective criterium remain. As such, the same patient might receive different “standard” cares dependent on the choice of clinicians. To remedy this, many standard healthcare guidelines have started with criteria as objective as possible. The same situation applies when attempting to establish cancer risk predictors.

With the advance in computer technology, many options are now available for us to evaluate patients in an objective manner. It is our duty to utilize these updated resources to optimize the care of our patient. One such technology is the algorithms generated by artificial intelligence in recent decades [11]. These algorithms enable detailed image recognition and data acquisition previously impossible by human readings. As a result, the development of these algorithms enabled a new set of standardized metrics that can help evaluate our patients’ conditions. With these tools, we can expand the basic criteria by including more objective measurements to establish better clinical guidelines, so as to improve patient care.

In this study, the incidence of HCC was slightly higher than expected because a large proportion of these cirrhotic patients had liver nodules under careful ultrasound/CT scan survey. The trabecular BD digitally assessed by CT scan was found to be a predictor for HCC development. Identification of such a group of high-risk patients is critical for physician practicing HCC surveillance, as many cirrhotic patients receiving CT examination are under high suspicion of developing small HCCs. Careful and close monitoring of this group of patients with a shorter interval may help in reducing the neglection caused by possible false negative imaging results or tumors that are still too small to be visualized. Although BD usually reduced with age, and age was a predictor for HCC, our present data showed that BD assessed by HU at L5 was a predictor better than age for HCC development from cirrhotic patients. Notably femur BD was not a predictor, suggesting that a specific area, but not any areas, for BD assessment was needed. The L5 BD assessed by CT, although significantly associated with HCC risk, was not suitable to stand out as an additional test for HCC surveillance, as CT was an expensive procedure, and the positive predictive value of this test was low. However, for cirrhotic patients receiving CT studies to study nodules, L5 BD could serve as a ready reference (simply check the HU from CT images). If no HCC characteristics was present in the CT study but the L5 BD was low, it would be advisable to perform an additional study.

Finally, another novel and intriguing finding in this study was that in HCC patients, the trabecular BD decreased rapidly right before HCC emerged, albeit there existed a background slow BD decreasing rate with age. This observation argued that BD loss was indeed directly associated with HCC development, but not indirectly linked to HCC through age. However, unless a CT scan could be performed very frequently, the feature of rapid BD decline could not be found. In Taiwan, however, liver ultrasound was performed for cirrhosis patients every 3 months, and if suspected nodules were detected, dynamic CT was ordered. Therefore, in areas where similar practice was performed, rapid decline of BD could possibly be noticed. 

A conceivable common factor for both HCC development and BD loss is vitamin D deficiency. Previous studies have indicated that vitamin D deficiency played a role in increased HCC risk [12,13,14]. Furthermore, recent studies suggested that vitamin D supplement had possible therapeutic roles in HCC [15,16,17]. Our present results were consistent with these notions.

There were some limitations for this study. Firstly, this was a retrospective instead of prospective verification study. Secondly, the information of calcium, vitamin D and bone formation marker was missing for further analysis. Thirdly, the clinical practice for HCC surveillance was different among different countries. In areas where a CT scan was not affordable for the general population, or the surveillance was not frequent enough, the usefulness of this study was limited. 

## 5. Conclusions

In summary, a verification study was conducted, which confirmed that BD was a predictor for HCC development in cirrhotic patients. Specifically, we showed that BD assessed by HU at L5 was the most significant independent predictor. Furthermore, a rapid decrease in BD could serve as an alarming sign for HCC development.

## Figures and Tables

**Table 1:** Baseline clinical data for 347 cirrhotic patients with CT scan records.

Variables	Value
Sex, male, %	234 (67.4%)
Age, years, mean ± SD	58.9 ± 11.3
Child-Pugh classification, A/B/C, %	305/28/14 (87.9%/8.1%/4.0%)
HBsAg, positive, %	222 (64.0%)
Anti-HCV, positive, %	89 (25.6%)
Alcoholism, yes, %	99 (28.5%)
AST, U/L, mean ± SD	51.6 ± 71.2
ALT, U/L, mean ± SD	49.4 ± 93.6
AFP, ng/mL, median (range)	3.3 (<2, 416.0)
WBC, ×10^9^/L, mean ± SD ^a^	5.9 ± 2.9
Hemoglobin, g/dL, mean ± SD ^a^	13.3 ± 2.8
Platelet, ×10^9^/L, mean ± SD ^a^	129.7 ± 67.0
T12 BD, HU, mean ± SD	175.3 ± 43.9
L5 BD, HU, mean ± SD	172.8 ± 48.8
Femur BD, HU, mean ± SD ^a^	91.1 ± 42.8
HCC, yes, %	93 (26.8%)
Follow up period, months, median (range)	52.3 (3, 223.4)

CT, computed tomography; SD, standard deviation; HCV, hepatitis C virus; AST, aspartate transaminases; ALT, alanine transaminase; AFP, alpha-fetoprotein; WBC, white blood cell; BD, bone density; HU, Hounsfield Unit; HCC, hepatocellular carcinoma. ^a^ Missing data for WBC, Hemoglobin, Platelet and Femur BD were noted in 113, 116, 112, and 4 patients, respectively.

**Table 2:** Linear regression analysis between T12 bone densities and L5 bone densities, Femur bone densities, and age.

Variables	Coefficient (95% CI)	*p*
L5	0.742 (0.688, 0.796)	<0.001
Femur	0.558 (0.467, 0.650)	<0.001
Age	−2.248 (−2.586, −1.910)	<0.001

CI, confidence interval.

**Table 3:** The correlations between Age, T12, L5 and Femur.

		Age	T12	L5	Femur
Age	Pearson correlation	1	−0.576 **	−0.562 **	−0.304 **
	Sig. (2-tailed)		0.000	0.000	0.000
	N	347	347	347	343
T12	Pearson correlation	−0.576 **	1	0.825 **	0.544 **
	Sig. (2-tailed)	0.000		0.000	0.000
	N	347	347	347	343
L5	Pearson correlation	−0.562 **	0.825 **	1	0.570 **
	Sig. (2-tailed)	0.000	0.000		0.000
	N	347	347	347	343
Femur	Pearson correlation	−0.304 **	0.544 **	0.570 **	1
	Sig. (2-tailed)	0.000	0.000	0.000	
	N	343	343	343	343

**, Correlation is significant at the 0.01 level (2-tailed).

**Table 4:** Cox proportional hazard analysis for clinical factors in relationship to time-to-HCC occurrence.

Variables	HR (95% CI)	*p*
Sex, male = 1	0.771 (0.508, 1.171)	0.223
Age, per year	1.023 (1.004, 1.042)	**0.017**
Child-Pugh classification, class B/C = 1	1.090 (0.527, 2.255)	0.816
HBsAg, positive = 1	0.693 (0.454, 1.059)	0.090
Anti-HCV, positive = 1	1.116 (0.709, 1.757)	0.634
Alcoholism, yes = 1	1.188 (0.754, 1.871)	0.458
AST, per U/L	1.001 (0.999, 1.003)	0.218
ALT per U/L	1.000 (0.998, 1.002)	0.923
AFP, per ng/mL	1.002 (0.997, 1.007)	0.435
WBC, per ×10^9^/L	0.988 (0.897, 1.089)	0.809
Hemoglobin, per g/dL	0.952 (0.846, 1.071)	0.412
Platelet, per ×10^9^/L	0.997 (0.993, 1.002)	0.253
T12 BD, per HU	0.994 (0.989, 0.999)	**0.013**
L5 BD, per HU	0.993 (0.989, 0.998)	**0.005**
Femur BD, per HU	0.996 (0.992, 1.001)	0.123
Multivariate analysis, stepwise mode ^a^		
L5 BD, per HU	0.994 (0.989, 0.998)	**0.007**
Multivariate analysis, entry mode ^b^		
Age, per year	1.010 (0.987, 1.033)	0.396
L5 BD, per HU	0.995 (0.989, 1.001)	0.077
Multivariate analysis, entry mode ^b^		
Age, per year	1.010 (0.988, 1.032)	0.371
L5 BD HU less than 161 = 1	1.676 (1.022, 2.748)	**0.041**

HBsAg, hepatitis B virus surface antigen; anti-HCV, antibody against hepatitis C virus; AST, aspartate transaminase; ALT, alanine transaminase; AFP, alpha-fetoprotein; WBC, white blood cell; BD, bone density; HU, Hounsfield unit; HCC, hepatocellular carcinoma. ^a^ Multivariate Cox proportional hazard analysis was conducted by including all variables and stepwise forward mode was applied. Probability for stepwise: entry, 0.05; removal, 0.10. ^b^ Multivariate Cox proportional hazard analysis was conducted by including age and L5 BD, and enter mode was applied. Bold, *p* < 0.05.

## Data Availability

Not applicable.
